# Quality of Care for Patients With Multiple Sclerosis—A Review of Existing Quality Indicators

**DOI:** 10.3389/fneur.2021.708723

**Published:** 2021-08-05

**Authors:** Anna Kristina Kraft, Klaus Berger

**Affiliations:** Institute of Epidemiology and Social Medicine, University of Münster, Münster, Germany

**Keywords:** multiple sclerosis, multiple sclerosis/therapy, quality improvement, quality indicators, health care, quality of health care

## Abstract

**Background:** The care of patients with multiple sclerosis (MS) calls for a lifelong guidance and treatment and results in a high resource utilization. Therefore, strategies for the assessment and improvement of the care process are crucial. Quality indicators have become a widely used instrument to determine quality in many areas of the healthcare system. The currently available sets of indicators for the quality of MS care are summarized in this review.

**Methods:** A literature search was conducted for reports that include statements on quality indicators for the care of people with MS. For the determination of the strength of the underlying evidence of the identified publications appropriate criteria of the PRISMA and AGREE-Statements were used. A further prioritization of the eligible indicators was based on the internal grading by the initial authors.

**Results:** Of the 465 included records in the search, 6 sources were finally identified, 3 demonstrating a high and the others a medium strength of evidence. In total, these six reports described 226 quality indicators for the treatment of MS. Of them, 147 were further included in the assessment due to the scope of this article. Among the 101 indicators that originated from reports with a high strength of evidence, 6 also had a high initial internal grading. These six identified quality indicators describe five important characteristics of a high-quality care of MS.

**Conclusion:** The search led to a scientifically evident set of six quality indicators for the assessment of care for patients with MS. These should be seen as starting points in the development of comprehensive sets of quality indicators in MS that addresses the individual objective of their use.

## Introduction

Healthcare systems intend to serve the population and provide quality care and the necessary structure and guidance for all involved stakeholders. The assessment of potential inadequacies and the continuing emphasis on the need of improvement are important prerequisites of modern healthcare systems (1). The definition of a best practice and the development of methods for the assessment and monitoring of quality healthcare are therefore inevitable (1, 2). While quality of care has various definitions depending on perspectives, priorities, and perceptions, the main components of the description typically include effective, safe, people-centered, timely, equitable, integrated, and efficient (1).

The quality of healthcare systems can be assessed or monitored by the use of indicators, which describe the appropriate performance, outcome, or structural characteristics as a result of an evidence-based standard of care (2, 3). By applying indicators, gaps in care quality can be identified, monitored, and often quantified, thus, providing the basis for improvements. Simultaneously, this allows comparisons between different providers, as well as the possibility to hold them accountable (3). There are numerous examples of chronic or acute conditions where consensus sets of established quality indicators are widely used for monitoring and improving the care for patients (4, 5).

The diagnosis of multiple sclerosis (MS) usually requires a lifelong treatment and causes high rates of resource utilization within each healthcare system. Due to the wide variety of possible symptoms, the management of the disease is very challenging and requires a multidisciplinary approach (6). These particular requirements aggravate the development of a set of quality indicators for the treatment of patients with MS. While several guidelines or recommendations have been published describing appropriate care, only a few indicators exist.

Given these shortcomings, the overall aim was to provide a structured review of published recommendations and statements for the treatment of MS that can serve for the purpose of being quality indicators. We concentrated on the possible impact on a broad variety of patient contacts and aimed for a high feasibility of the integration and consideration of the described results in the care of every patient with MS. Special patient circumstances like pregnancy, time of first diagnosis of MS, change of treatment, or disease-modifying therapy (DMT)-specific recommendations were not included.

## Methods

**Figure 1:**
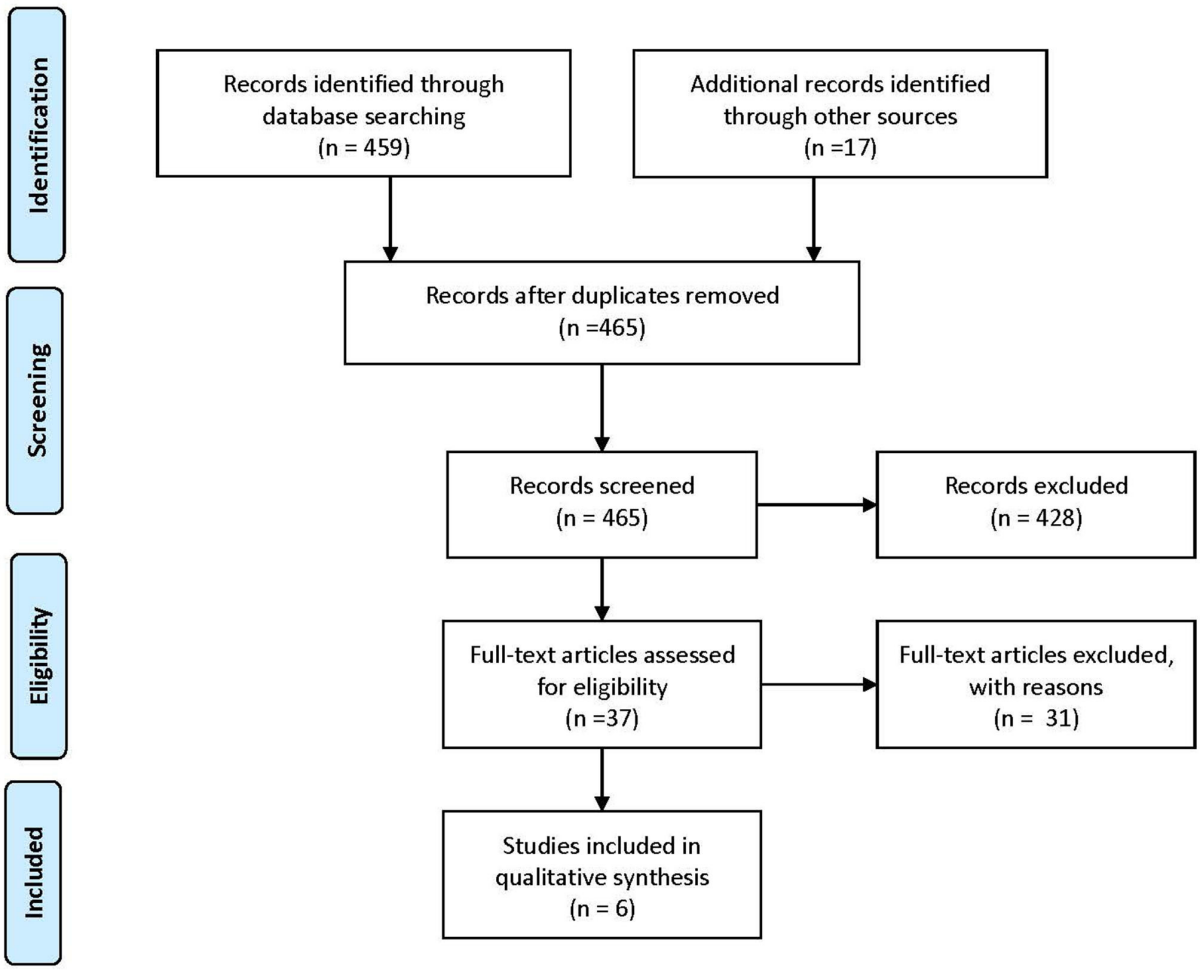
PRISMA-Flowchart (7) of the conducted literature search.

This review is based on publications that include statements, recommendations, or guidelines for the quality of care assessment for patients in different stages of MS. For the identification of eligible publications, a literature search was conducted in PubMed in January 2021 using the following MeSH Terms: “Multiple Sclerosis,” “Quality Indicators, Health Care,” “Total Quality Management,” “Quality Assurance, Health Care,” “Quality Improvement.” The search identified 459 eligible records for this review through the database search and 17 through other sources (Figure 1).

From the total of 465 identified publications, 428 records were excluded for not being within the previous described scope of this review after screening their abstracts. The full text was accessed for the remaining 37 articles, among them, 6 reports included quality indicators. All studies, regardless of their objective or nature, that had an MS care scope and defined recommendations, quality indicators, or content related to assessing a high quality of care were included. These sources were processed in two steps. First, the strength of evidence for each study was assessed by the evaluation of its formal quality as a surrogate and sorted in three categories. Publications that reached a high strength of evidence met all or almost all stated criteria. In a second step, we assigned the internal rating (Levels A, B, and C) of the original publication to the included indicators.

The strength of the evidence was assessed using a combined set of criteria of the well-established PRISMA and AGREE-Statements (7, 8). Due to their different thematic focuses, items from the PRISMA-Statement were primarily utilized to assess the formal quality of the systematic review, while the AGREE-Statement was applied for the assessment of the indicator or recommendation development panel and the performed development process for the indicators. The assessment of the formal quality of the literature review in each report was based on the items “Information source,” “Search,” “Study selection,” and “Study characteristics” from the PRISMA-Statement. For the assessment of the structure of the panel, which developed the recommendations, the items “Group membership,” “Founding Body,” and” Competing interests” were used. Because the AGREE-Statement did not strongly focus on a well-balanced structure and appropriateness of the development panel, the authors added items for assessing the diversity of the panel and their ability to represent all major physician and non-physician stakeholders (e.g., neurologists, patient representatives, nurse practitioners, rehabilitation specialists, occupational therapist, physical therapist, and psychiatrist) in the therapy of MS. By using the statements on “Formulation of Recommendations,” “Link Between Recommendation and Evidence,” “External Review,” “Specific and Unambiguous Recommendations,” and “Identifiable Key Recommendations,” the development process of the items, and its transparency and suitability were evaluated. If companion sources were used to determine the strength of evidence in the assessment, it is shown in Table 1. While combining the results of the three categories, we emphasized more on the formal quality of the review than on the other two. Next, we excluded all statements concerning special circumstances or medication and concentrated on statements concerning the care routine. To be able to further prioritize the identified indicators, we used the internal categories provided by the individual authors, if existing, and standardized them to Levels A (being the best), B, and C.

**Table 1:** Summary of assessment of formal quality of the identified publications.

**Paper**	**Assessment of formal quality of the review**	**Assessment of structure of panel**	**Assessment of development of process**	**Evidence strength**
Hobart et al. (9)^*^	Low	High	High	Medium
Cheng et al. (10)	Medium	High	High	Medium
Rea-Grant et al. (11)^**^	High	High	High	High
Rae-Grant et al. (12)	Low	High	High	Medium
National Institute for Health and Care Excellence (13)	High	High	High	High
Montalban et al. (14)	High	Low	High	High

* *Including companion sources (15)*.

** *including companion sources (16)*.

## Results

The literature review resulted in a final list of six publications that include quality indicators for the treatment of MS. Two of the included publications were mainly developed as guidelines for the treatment of MS (13, 14), while the others (9–12) concentrated on the development of instruments for the improvement of the treatment quality.

Montalban et al. developed the ECTRIM/EAN guideline for the pharmacological treatment of people with MS (14). This publication has a high rating for the formal quality of the review as well as for the development process. Due to its nature as a guideline of a scientific organization, it was developed by a very homogenous group of experts (experienced physicians) and, therefore, only achieved a low rating for the structure of the panel due to its lack of diversity. This circumstance was considered as minor by the authors, and therefore, the publication achieved a high overall rating. The British National Institute for Health and Care Excellence (NICE) developed the clinical guideline for the management of MS in adults (13). With regard to the defined criteria, this work exhibited a high quality in all three categories. It was developed by a multidisciplinary group that consisted of professionals as well as lay members and represented a high transparency and quality regarding its included literature review as well as the structure of the development process. The publications for the AAN (American Academy of Neurology) by Rae-Grant et al. (11, 12) were both generated by a well-balanced research group (physicians, patients, nurses, and other stakeholders) and followed a high-quality development process. Their work from 2018 is based on a systematic review that was also published in a companion article (16); the work from 2015 provided very little information about the literature foundation, which resulted in a low rating in this category. The work by Hobart et al. (9) emphasizes on a diverse structure of the development panel and an appropriate process of developing indicators for good quality care for patients with MS. The authors gave no information about the used scientific foundation, thus, resulting in a low rating in this category. The publication by Cheng et al. (10) is the oldest developed set of indicators. It provides only limited information about the used literature, but has detailed information on the diverse development group and the applied process.

The six included articles contained in total 226 statements. Of these indicators, 33 were developed for the time of diagnosis, 38 for the treatment with a specific DMT and 8 for specific circumstances, e.g., pregnancy and childbirth. Therefore, these were not suitable for the purpose of this paper that focuses on the feasibility of the integration of the described results in a broad variety of patient contacts during the management of MS. This left a set of 147 indicators for further consideration. A full listing can be found in the supplement.

For further evaluation of the quality indicators, we categorized them according to the framework proposed by Donabedian in “structure,” “process,” and “outcome” (2). This categorization led to a total of 9 statements indicating the quality in terms of the outcome, 95 related to the process in healthcare, and 8 referred to the structural setting. For the remaining 35 indicators, a clear distinction between process and structure was not feasible.

As shown in Table 2, the three articles which were ranked with a high quality of evidence described 101 statements, six of them Level A, 27 indicators having Level B, and 11 having Level C. Fifty seven indicators have not been ranked in their initial article. The remaining articles, which had a medium quality of evidence, included 59 indicators, which did not have any initial internal ranking system.

**Table 2:** Number of indicators that are included in the selected sources.

	**Level A**	**Level B**	**Level C**	**NA**	**Sum**
**High**	**6**	**27**	**11**	**57**	**101**
Montalban et al. (14)	1	3	8		12
NICE (CG186) (13)	2			57	59
Rae-Grant et al. (11)	3	24	3		30
**Medium**				**46**	**46**
Cheng et al. (10)				21	21
Hobart et al. (9)				16	16
Rae-Grant et al. (12)				9	9
**Sum**	**6**	**27**	**11**	**103**	**147**

*The sources are ranked according to the strength of the underlying evidence and the indicators according to the internal grading (Levels A–C) by the initial authors*.

The six statements with the highest evidence quality and the highest internal quality cover five important characteristics of MS-management (Table 3). These contain the need to include a reporting process in case of new or worsening of symptoms and the need for a coordinated multidisciplinary approach. In addition, the promotion of appropriate physical exercise and of an active cooperation between physician and patient in the decision process for a treatment with DMTs, as well as the early onset of DMTs, was highlighted.

**Table 3:** Selected indicators and their characteristic of management of MS.

**No**	**Indicator**	**Described characteristic of management of MS**
1	“Clinicians must counsel people with MS on DMTs to notify the clinicians of new or worsening symptoms” (11).	Reporting process in case of new or worsening of symptoms
2	“Consider supervised exercise programmes involving moderate progressive resistance training and aerobic exercise to treat people with MS who have mobility problems and/or fatigue” (13).	Promotion of appropriate physical exercise
3	“Care for people with MS using a coordinated multidisciplinary approach. Involve professionals who can best meet the needs of the person with MS and who have expertise in managing MS including consultant neurologists, MS nurses, physiotherapists and occupational therapists, speech and language therapists, psychologists, dietitians, social care and continence specialists, and GPs” (13).	Need for a coordinated multidisciplinary approach
4	“Clinicians must ascertain and incorporate/review preferences in terms of safety, route of administration, lifestyle, cost, efficacy, common adverse effects (AEs), and tolerability in the choice of DMT in people with MS being considered for DMT” (11).	Active cooperation between physician and patient in the decision process
5	“Clinicians must engage in an ongoing dialog regarding treatment decisions throughout the disease course with people with MS” (11).	
6	“Offer early treatment with DMDs to patients with active RRMS as defined by clinical relapses and/or MRI activity (active lesions–contrast-enhancing lesions; new or unequivocally enlarging T2 lesions assessed at least annually). Also includes CIS fulfilling current diagnostic criteria for MS” (14).	Early onset of DMT

*MS, multiple sclerosis; DMT, disease-modifying therapies*.

Indicator No. 1, developed by Rae-Grant et al. (11), actively encourages physicians to educate the patient to report new or worsening symptoms if they are treated with a DMT. Despite using different terminology, this indicator is included in each of the six assessed publications, thus, exhibiting a strong backing in the literature. Moreover, it addresses the requirement of an active engagement of the patient by the physician to take an active role in their care. Indicator No. 2 suggests the consideration of exercise programs for treating mobility problems and fatigue. Many other sets consider not only a positive effect of appropriate physical activity to individual symptoms but to the overall course of MS and the overall physical and mental health (10, 12, 13). Indicator No. 3 highlights the necessity for a diverse multidisciplinary group of healthcare professionals in the treatment of people with MS, as well as their active cooperation. This requirement can also be found in numerous other indicator sets (9–11, 13) and is often associated with forwarding information to other professionals in case of relapses (13) or the active referring to specialists in case of symptom management (10, 13, 17). The listing of professions to be involved shows that this indicator does not only focus on the care for somatic conditions but also for psychological and social care. Two of the selected indicators (Nos. 4 and 5) highlight the importance of an active cooperation by the patient during the course of the disease. One of the terms concentrates specifically on the choice of treatment for eligible patients, while the other insists on an active cooperation by all patients with MS during the entire course of the disease. Both indicators have in common that the physician has an active role in reviewing preferences, creating a dialog, and engaging the patient constantly. Similar indicators in the reviewed publication sets ask for a cooperation of the physician and the patient during the decision for treatment of relapses or symptoms (10, 13) and other specific occasions. The last chosen indicator (No. 6) requests for an early treatment start with DMTs for patients with active RRMS. Additionally, it includes a definition of clinical criteria as well as a timeline for MRI as a diagnostic test. Information about diagnostic criteria, treatment start, and a timeline for monitoring the course of the disease is included in many of the assessed indicator sets (9, 11, 13).

## Discussion

In this paper, we reviewed the current available literature of developed sets of quality indicators for the treatment of patients with MS and ranked them according to appropriate described quality criteria. We concentrated on the respective sources and their applied internal grading system to identify the highest scoring indicators. This process led to a set of six indicators, describing five characteristics for the assessment of a high quality of care in patients with MS. These cover the participation of patients in their care process, the monitoring of symptoms and possible side effects, the encouragement of physical activity, the implementation of multidisciplinary health care, and the initiation of DMT for the treatment of MS.

Indicator No. 1 aims toward monitoring of effects, potential side effects, and the course of the disease, and an active engagement of the patient by the physician. Additionally, it requests the development of a structured process [e.g., management plan or point of contact (10, 13)] in case of a relapse, disease progression, or other important events. Beside these components, important issues like a specific timeline for the monitoring of the treatment with diagnostic tools (e.g., MRI-imaging) are not included here, but can be found in other indicators by various authors (9, 11, 13, 14). An additional identified indicator (No. 3) highlights a coordinated multidisciplinary approach for the care of people with MS, including the wide variety of appropriate non-medical professions who should be included in the management of the disease. The implementation of this indicator highly depends on the structure of the healthcare system and established processes in the local care process. Indicator Nos. 4 and 5 focus on an active role of the patient in their care; most of the assessed indicator sets included recommendations for that, some even specifying specific circumstances. According to the indicator, the physician has a constant active part in engaging the patient, even if she or he previously rejected to be involved in decisions about the treatment. Finally, the positive effect of appropriate exercise for people with MS is widely acknowledged (17) and addressed in Indicator No. 2. Evidence for the appropriate exercise intervention for patients with MS has also been the topic of a current meta-analysis (17).

Because of concentrating on the formal quality of the literature and the initial internal ranking, the resulting indicators represent high relevance, but do not cover all important components of a high quality treatment of MS. The set has to be extended to cover the entire treatment spectrum for people with MS. There are many more quality indicators in connected disease areas, which are not included in this review but may have important implications on the quality of care for people with MS. The further improvement of the diagnostic methods, criteria, and therapeutic options for the management of MS will not only influence the development of any new indicator but also the evaluation of existing indicators. Despite the use of PubMed as a single main data source, we do not expect beneficial publications in other sources.

So far, only a few studies have used indicators for the assessment of the quality of the treatment of MS (18–20). They all used secondary data to assess the quality of the management of MS-related symptoms (depression, spasticity, fatigue, and mobility impairment/falls), as well-appropriate timelines for the onset of DMT and the discussion of the diagnosis with the patient. Results on the effect of a quality indicator-based care process on the course of disease in MS patients are scarce. The fact that there is only a small amount of publications in a field that is crucial for the improvement of the therapy of patients with MS indicates the need for further studies. Thus, our review recommends the development of additional sets of indicators, complementing those summarized here with an individual prioritization of the previous stated components of quality care by the WHO in mind. Subsequently, new sets of indicators must be tested and evaluated in the real-life setting to show the possibility of generating a positive impact for the people with MS.

## Data Availability Statement

The original contributions presented in the study are included in the article/Supplementary Material, further inquiries can be directed to the corresponding author/s.

## Author Contributions

AKK and KB analyzed the data, and contributed to and approved the final manuscript. AKK prepared the first draft of the manuscript. All authors contributed to the article and approved the submitted version.

## Conflict of Interest

KB has received within the last 10 years investigator-initiated research funding as principal or coordinating investigator in the areas of stroke, depression and subclinical atherosclerosis, multimorbidity and health services research, diabetes, multiple sclerosis and for the German Nako Health Cohort from the German Ministry of Research and Technology (BMBF). For the conduction of an investigator initiated clinical register for multiple sclerosis he has received a grant from the German Competence Net Multiple Sclerosis with funds from Biogen Idec. The remaining author declares that the research was conducted in the absence of any commercial or financial relationships that could be construed as a potential conflict of interest.

## Publisher's Note

All claims expressed in this article are solely those of the authors and do not necessarily represent those of their affiliated organizations, or those of the publisher, the editors and the reviewers. Any product that may be evaluated in this article, or claim that may be made by its manufacturer, is not guaranteed or endorsed by the publisher.
